# Sleep Quality and Sleep Behaviors in Varsity Athletes: A Pilot Study

**DOI:** 10.3389/fspor.2022.906663

**Published:** 2022-06-22

**Authors:** Lyndon J. Rebello, Andrew W. Roberts, Alyssa M. Fenuta, Anita T. Cote, Michael E. Bodner

**Affiliations:** School of Human Kinetics, Trinity Western University, Langley, BC, Canada

**Keywords:** sleep behaviors, sleep quality, university athletes, ASSQ, ASBQ

## Abstract

Sleep hygiene practices may hinder university athletes from obtaining quality sleep to support health and performance. We sought to provide a comprehensive evaluation of sleep quality and behaviors in varsity athletes using validated sleep questionnaires: the Athlete Sleep Screening Questionnaire (ASSQ) and the Athlete Sleep Behavior Questionnaire (ASBQ). Sixty-four (*n* = 64) athletes participated (54% female; 71% Caucasian). The mean age was 20.3 ± 1.7 years and the mean BMI was 23.3 ± 3.3. Fifty-one percent met the threshold for adequate sleep (7+ h) and 54% reported being somewhat/very satisfied with sleep quality. Global scores for ASSQ Sleep Difficulty and ASBQ sleep behaviors were significantly correlated (*r* = 0.31; *p* = 0.014) and not significantly different across age, academic year, or residence. According to the ASSQ, 11% and 24% were classified as having severe or moderate sleep problems, respectively. The ASBQ categorized 62% as having “poor” sleep behaviors. Notable sleep-influencing factors included a high frequency of emotional/cognitive processing of sport-performance issues (46.9%), frequent use of light-emitting devices before bed (90%), training after 7 pm (65%), and the use of sleep medication (19%). Half of the university athletes did not meet the thresholds for adequate sleep, and some may require a referral for clinical sleep issues. The majority of these athletes' sleep behaviors do not promote adequate sleep. The ASSQ shows utility to assess gradations in clinical sleep difficulty; the ASBQ could be used in concert with the ASSQ to discern “cognitive and physiological arousal” targets for use in educational workshops designed to promote optimal sleep hygiene in university athletes.

## Introduction

Sleep is a requisite for the attainment, maintenance, and restoration of physical and mental health, including physiological function, cognition, and physical recovery (Mah et al., 2018; Walsh et al., 2021). As such, sleep is vital for university athletes, contributing not only to recovery from physical stresses arising from training schedules and competition but also to academic success via learning and cognition (Okano et al., 2019).

The National Sleep Foundation recommends that young adults (18–25 years) attain between 7 and 9 h of sleep each night (Hirshkowitz et al., 2015). Findings from a sample of elite athletes from multiple sports revealed that they needed ~8.3 h sleep per night to feel rested, but only achieved 6.7 h on average (Sargent et al., 2021). The negative health effects associated with chronic inadequate sleep include cardiovascular disease, diabetes, and obesity (Luyster et al., 2012; Ramar et al., 2021). Sleep disturbances also impair adaptive immunity leading to increased susceptibility to diseases and may be linked to negative psychological consequences in young athletes (Fullagar et al., 2015; Irwin, 2015; Fox et al., 2020; Grandner et al., 2021). Specific to athletic success, insufficient sleep is also associated with lower psychomotor performance including slower reaction times, concentration, attention, and speed processing, all of which may diminish competitive performances (Fullagar et al., 2015; Monleon et al., 2018; Fox et al., 2020; Grandner et al., 2021).

The quantity and quality of sleep may be predicated based on a person's sleep hygiene: engaging in behaviors that promote good sleep (e.g., regular, relaxing bedtime routine) and avoiding behaviors that interfere with sleep (e.g., consuming caffeine in the hours shortly before bed) (Irish et al., 2015; Walsh et al., 2021). However, engagement in university life brings with it a series of challenges including new social contexts (in some cases sleep-disruptive living arrangements, e.g., roommates, room temperature, noise), demanding academic workload, and financial stresses (e.g., part-time work), all of which can increase stress levels and some of which can affect sleep hygiene and sleep itself (Brown et al., 2017; Amaral et al., 2018). Further, it is believed varsity student athletes in particular may be further predisposed to poor sleep hygiene behaviors and sleep quality, in part due to the additional stresses of travel and adjustments to time zones (Fox et al., 2020).

The prevalence of behavioral factors that influence sleep quality and quantity are understudied in the university athlete population using athlete-specific questionnaires. Therefore, the purpose of this pilot study was to provide a cross-sectional descriptive assessment of sleep behaviors and sleep characteristics of male and female varsity athletes using two validated sleep questionnaires tailored specifically for athletes.

## Materials and Methods

This study was approved by the University Human Research Ethics Board, and all participants provided written informed consent. Varsity male and female athletes from Trinity Western University (Langley, British Columbia, Canada) were recruited over two time periods: February 2020 and January 2022 to complete general demographic questions, anthropometric measurements (i.e., height, weight; Seca GmbH, Hamburg, Germany), and two athlete-specific sleep questionnaires, the Athlete Sleep Screening Questionnaire (ASSQ) and the Athlete Sleep Behavior Questionnaire (ASBQ) (Samuels et al., 2015; Driller et al., 2018). The ASSQ is a quantitative clinical sleep screening tool that assesses the quality, length, and consistency of sleep to provide a global Sleep Difficulty Score (SDS) for athletes. The SDS is derived from five Likert-scale questions that are summed; a higher SDS indicates a greater likelihood of a clinical sleep problem using the following cut-off points to delineate severity: none (0–4), mild (5–7), moderate (8–10), and severe (11–17) (Bender et al., 2018). The ASSQ also assesses sleep modifiers including travel, chronotype (morning or evening type), and sleep disordered breathing (e.g., apnea). The ASSQ provides interventions based on SDS scores and modifiers.

The ASBQ assesses the sleep hygiene habits of athletes and provides recommendations if needed. The ASBQ queries how often athletes engaged in specific sleep and sport behaviors in the past month and ranks their responses using a 5-point Likert scale. A global score ranging from 18–90 is calculated from the sum of these responses (Driller et al., 2018). A higher global score indicates a greater likelihood of poorer sleep-related behaviors; the authors of the ASBQ suggest a global score ≤ 36 equates to “good” sleep behavior, 37-41 “moderate” sleep behavior, and ≥42 = “poor” sleep behavior (Driller et al., 2018). The ASSQ has shown moderate internal consistency (r = 0.74) and strong test–retest reliability (r = 0.84); the sensitivity of the ASSQ to detect clinically meaningful sleep problems is reported to be 0.81 in accordance with sleep medicine physician ratings (Bender et al., 2018). The ASBQ has shown small/moderate correlations with other sleep questionnaires and strong test–retest reliability (Driller et al., 2018).

Descriptive statistics (frequencies, percentages, means, and standard deviations) characterized participants' sociodemographic data and ASSQ and ASBQ scoring. Global score distribution for ASSQ SDS and ASBQ were assessed for normality using the Shapiro–Wilks test. Associations between variables were derived using Spearman and Pearson correlation. Chi-square assessed any non-parametric associations. Independent *t*-tests with Bonferroni correction assessed differences for ASSQ SDS and ASBQ global scores across sex and residency (on- or off-campus); one-way ANOVA assessed the said differences across the academic year. Statistical significance was set at *p* < 0.05.

## Results

Sixty-four individuals (29 males and 34 females) volunteered to participate; 57% lived on campus and almost half (46%) were in their first or second year of university. The mean age was 20.3 ± 1.7 years. A majority of participants in the study were identified as Caucasian (71%). See Table 1 for sociodemographics.

**Table 1:** Sociodemographics (*n* = 64).

	**%/mean (SD)**
**Sex**
Female	54
**Age**	20.3 (1.7)
**BMI**	23.3 (3.3)
**Ethnicity**
Caucasian	71
Southeast Asian	6
Black	5
South Asian	3
Mixed	11
First Nations	2
Other	2
**Sport**
Soccer	41
Rugby	17
Basketball	13
Volleyball	13
Track and Field/Cross-country	8
Hockey	8
**Phase of Season**
In-season	51
Off-season	40
Pre-season/Other	9
**Residence**
On-campus	57
Off-campus	43
**Academic Year**
First	22
Second	24
Third	21
Fourth	16
Fifth	17

Global scores of ASSQ SDS and ASBQ sleep behaviors were 6.5 ± 3.0 and 42.6 ± 5.4, respectively; both global scores were normally distributed. A moderately low association was noted between global ASSQ SDS and ASBQ scores (r = 0.31, *p* = 0.014). There were no significant differences in ASSQ SDS global scores or ASBQ global scores across gender, residency (on- or off-campus), or academic years (first year, second year, etc.). Altogether 37% of the participants were classified as having either a moderate or severe clinical sleep problem based on ASSQ SDS.

Approximately half (51%) of athletes met the minimum requirements of 7–9+ hours of sleep, and overall 54% reported that they were somewhat satisfied with their sleep. Quantity of sleep and sleep satisfaction were positively correlated (r = 0.48; *p* < 0.001). ASSQ characterized 84% of respondents as a “morning person” chronotype.

**Figure 1:**
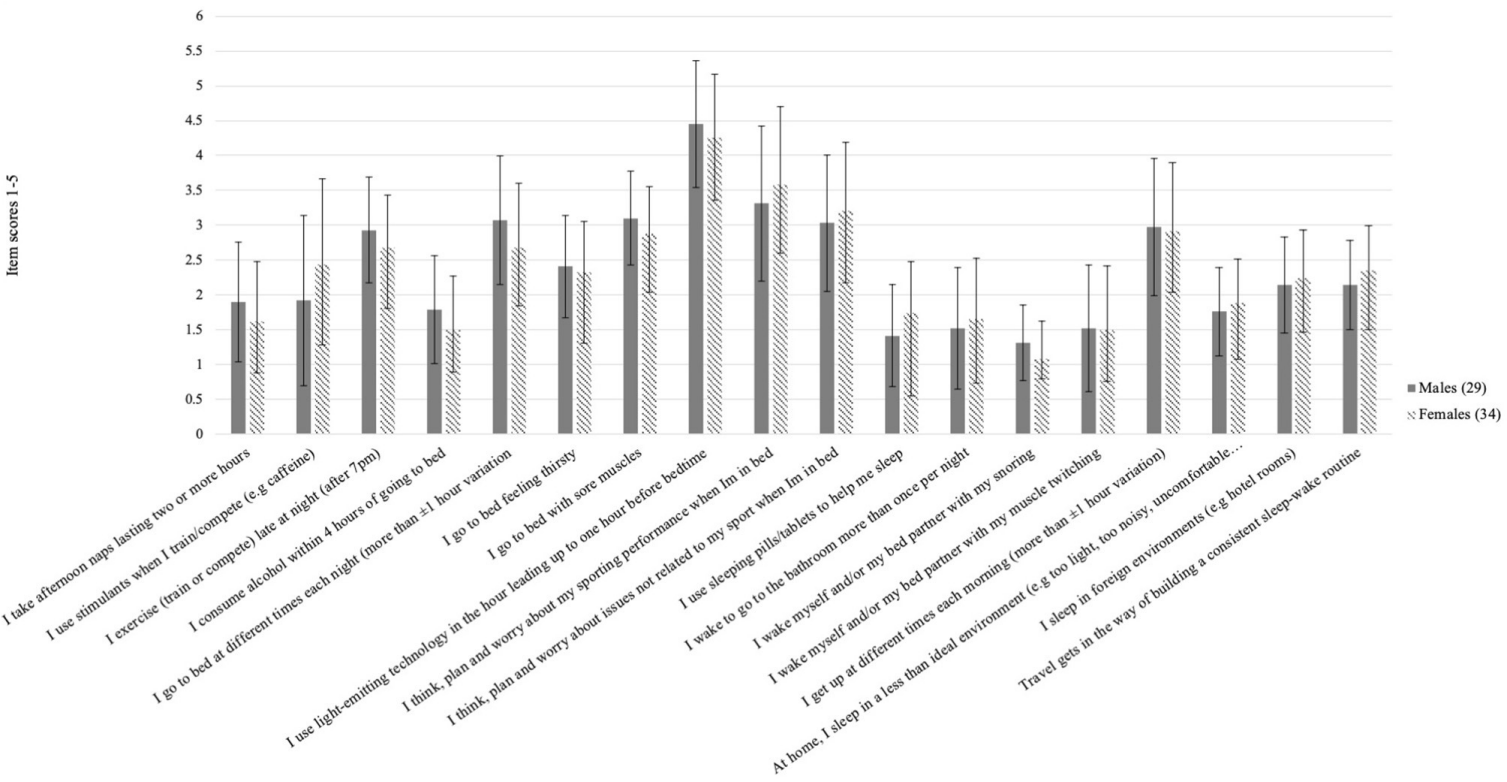
Mean scores and standard deviations for male (*n* = 29) and female (*n* = 34) athletes surveyed for each item of the 18-question Athlete Sleep Behavior Questionnaire (ASBQ).

The ASBQ characterized 62% of participants as having “poor sleep behaviors.” Figure 1 highlights the mean scores of each item of the 18-question ASBQ. Table 2 highlights ASSQ SDS categorization, ASBQ sleep behavior characteristics, sleep quantity, and sleep quality. Notable behavioral characteristics related to sleep included a substantive proportion who reported frequent or persistent cognitive or emotional processing either related to sport performance (48%) or non-sport (30%) issues while trying to sleep. Greater frequency of cognitive/emotional processing related to sport was positively associated with more severe clinical sleep issues (r = 0.247, *p* = 0.013) and negatively associated with fewer hours of sleep per night (r = −0.224, *p* = 0.03) but not associated with sleep satisfaction. Cognitive/emotional processing related to non-sport issues was not associated with either clinical sleep problems or hours of sleep per night. There were no differences across gender for the frequency of sport-related processing.

**Table 2:** Proportional representation (%) of sleep hours, satisfaction, Athlete Sleep Screening Questionnaire SDS, and Athlete Sleep Behavior Questionnaire (ASBQ) sleep behavior categories (*n* = 64).

**Sleep (hours)**	**%**
8+	16
7–8	36
6–7	33
5–6	15
**Sleep satisfaction**	
Very satisfied	8
Somewhat satisfied	45
Neither satisfied/dissatisfied	14
Dissatisfied	25
Very dissatisfied	8
**ASSQ Sleep Difficulty Score Category**	
None	27
Mild	37
Moderate	25
Severe	11
**ASBQ Sleep Behavior Category**	
Good	19
Moderate	20
Poor	61

As indicated by both ASSQ and ASBQ, athletes engaged in frequent/daily use of electronic devices 1 h before bed (90%) and training or competing late at night (after 7 PM) sometimes or frequently (65%). Slightly over half of the athletes took naps only once or twice during the week (57%), with 11% napping three to four times per week; overall males napped more frequently than females *X*^2^ (2, *N* = 63) = 8.2, *p* = 0.012. In terms of pharmacological influences, a notable proportion (19%) reported using either prescribed or over-the-counter sleeping tablets/pills to help with sleep sometimes/frequently/always; of these 13% were female.

## Discussion

In this study, we described sleep quality and sleep behaviors in collegiate athletes using validated, athlete-specific measures. Similar to others, the findings of our pilot study show that sleep quantity, quality, and behaviors are suboptimal for many university athletes (Rabin et al., 2020). The limitations of this study include the use of self-reported assessments of sleep quality and behaviors (including recall bias) and the lack of the use of actigraphy to validate sleep hours. The use of cross-sectional design limits any assessment as to whether or not current sleep quality and behaviors have changed from what is shown in this report. Other factors that could have influenced sleep such as training volume and intensity and nutrition (meal timing) were not included in this analysis. The sample size in this study is relatively small for a survey study; however, the varsity athlete population is a fairly homogeneous group: same institution, narrow age range, and similar training environment with all dryland training in the same center, under the direction and guidance of the same strength and conditioning coach. While this strengthens the findings in this current pilot, moving forward greater representation is desirable for greater generalization.

Perhaps a key finding is that sleep difficulty (as indicated by ASSQ SDS global scores) and sleep behavioral issues (as indicated by ASBQ global scores) appeared to be consistent across gender, place of residence, and academic year. Driller et al. found no difference in ASBQ global scores across gender, as we have also shown (Driller et al., 2018). However, our findings contrast those of Rabin et al. who showed differences across genders, as well as differences between academic years (Rabin et al., 2020). These differences could be explained in part given the timing of assessments. Rabin assessed athletes enrolled in four National College Athletic Association schools (Division 1-3) prior to the start of the school year during annual physicals and practices, whereas our assessments took place in the middle of the academic year, in the midst of academic workloads (Rabin et al., 2020). Further, it is likely that our sample participants were exposed to somewhat greater environmental homogeneity that may have influenced the findings. These factors include attendance at the same relatively small institution, similar dryland training environment in the same center under the direction and guidance of the same strength and conditioning coach. It is plausible that some athletes shared similar social groups or courses together which could have impacted their sleeping behaviors (e.g., roommates, class study groups, peer groups). The influence of social connections has been suggested as a potential factor influencing sleep behaviors in professional athletes post-game; social connections may also be a factor in sleep behaviors of varsity athletes (Fullagar et al., 2016; Wang et al., 2021).

The global scores of both ASSQ SDS and ASBQ also showed a small association in our findings showing the linkage between sleep behaviors and clinical sleep issues. Our findings are similar to those of Knufinke who showed an association between general sleep hygiene and general sleep quality in elite athletes (Knufinke et al., 2018). The proportion of university athletes in our study characterized by the ASSQ as having moderate to severe clinical sleep problems (37%) was slightly higher compared to other findings in varsity athletes, but nevertheless further validates the scope of clinical sleep issues in this population (Rabin et al., 2020).

The global score for ASBQ in this study (42.6 ± 5.4) was comparable to similar athletic populations (43.5 ± 5.8) (Driller et al., 2018). Similar to Lastella et al., we found that quantity of sleep and sleep satisfaction showed a moderate positive association (Lastella et al., 2014). In terms of behaviors conducive to sleep, a substantive majority of the participants fell short of the standard of “good behaviors” as defined by the ASBQ. There were some notable findings in this regard, given the relatively small sample. First, for almost half of the athletes, it appears that a greater frequency of emotional/cognitive processing of issues related to their sport performance is associated with greater sleep difficulty and less total hours of sleep per night. It is conceivable that during pre-sleep, worries and excessive cogitation associated with sport performance or competition, especially if viewed negatively, could lead to increased autonomic arousal and emotional distress resulting in sleep disturbance (Harvey, 2002). An exploratory investigation of the psychological correlates of insomnia in professional soccer players showed that sport-related worry, rumination, and negative emotions were significant factors related to insomnia, mediated by pre-sleep arousal (Ballesio et al., 2021). The easing of psychological strain has been suggested as a target for improving sleep hygiene in elite athletes (Knufinke et al., 2018).

Second, a large proportion of university athletes used electronic devices within an hour before bed; this finding was unsurprising given the high prevalence of use in university students and elite athletes (Knufinke et al., 2018; Pham et al., 2021). Blue light emissions from personal electronic devices, such as computer screens, may interfere with circadian rhythms by delaying melatonin production and thus sleep initiation (Cajochen, 2011). There is some evidence to suggest that wearing amber lenses block blue light and the use of these may improve sleep (Buchart and Phelps, 2009; Shechter et al., 2018). However, engagement in screen time within a short time of retiring for sleep may also be associated with increased psychological arousal that delays sleep onset or occupies a part of the total time in bed that is meant for sleep, termed “sleep displacement' (Mauri et al., 2011; Bhat et al., 2018).

Third, a majority of athletes reported training or competition later in the evening on a regular basis. The effects of evening training and competition on sleep duration and quality are somewhat equivocal. Late evening training or competition provides an environmental context that lends itself to both increased wakefulness and a natural extension of later bedtime (Fullagar et al., 2016). In some cases, sleep duration was shown to be markedly reduced and sleep quality adversely affected following evening competitions in soccer and rugby athletes, especially when wake times were held constant (Fullagar et al., 2016; Nedelec et al., 2019; Conlan et al., 2021). However, others have shown that training in the evening has no effect on sleep quality in athletes (Myllymaki et al., 2011; Thomas et al., 2020).

Finally, a small proportion (19%) of athletes reported using sleep medication sometimes/frequently to help with attaining sleep, and a majority of those were female. Reported prevalence of prescription sleep aid use in collegiate athletes is about 3%; the use of specific over-the-counter sleep aids is less known, although Knufinke reported that melatonin use in a sample of elite athletes was 3% (Taylor et al., 2016; Knufinke et al., 2018; NCAA Research, 2018). Notwithstanding the inherent health risks of using pharmacological sleep aids, the use of prescription sleep medication in athletic populations is understudied in terms of their viability to improve sleep quality and quantity, their impact on sport performance, and impact on cognitive function during and post-performance (Taylor et al., 2016). While global scores related to sleep quality and behavior may not differ across gender, investigation of sub-factors such as the use of prescription medication to aid sleep in larger samples and across gender may be warranted.

Although the association between sleep behaviors and sleep difficulty remains to be more fully elucidated, our findings show “cognitive and physiological arousal” targets that could be addressed in varsity athletes to promote both the necessity of sleep and the optimization of sleep behaviors to potentiate sleep quantity and quality. Sleep workshops or training sessions could address these targets and be integrated at specific or strategic intervals throughout the academic or competitive season (e.g., pre-season, mid-season, and post-season) (Kaier et al., 2016; O'Donnell and Driller, 2017). Such workshops could provide education and instill self-efficacy for the adoption of sleep behaviors that promote quality sleep. This could include, for example, pragmatic factors related to screen time use by educating athletes to engage blue light filter options (on many smartphones) or the distribution of amber lenses paired with education to use them effectively, as well as avoiding stimulants and distractions before going to bed, and engaging in a consistent bedtime routine (Buchart and Phelps, 2009; Shechter et al., 2018; Vitale et al., 2019). The incorporation of mindfulness training into such workshops could be helpful for athletes who engage in frequent cognitive/emotional processing, particularly related to the appraisal of their sport involvement and performance (Kaier et al., 2016; O'Donnell and Driller, 2017). Mindfulness training has been shown to reduce rumination levels and smartphone addiction in university students, and in combination with sleep hygiene sessions, improve sleep quantity in elite junior tennis players (Cheng et al., 2020; Lever et al., 2021). However, in some cases of severe sleep disturbance (i.e., insomnia) a referral for cognitive behavior therapy would be appropriate (Halson, 2019). Some sleep-altering factors may be more challenging to adequately address. For example, daily practices and weekly competitions that extend to the early/late evening hours are often pre-determined by academic schedules, availability of facilities, and league schedule, and are outside the athlete's control. However, athletes could be counseled to select academic courses offered at times that allow for a greater time window for added sleep if practice scheduling is known ahead of time (i.e., late morning or early afternoon courses if late evening practices/competitions are frequent). Finally, although the proportion of athletes who use prescription/over-the-counter sleep medication is small, there could be room for the incorporation of sober discussion by team physicians related to the use and side effects of such sleep aids.

Although recommended by expert consensus, sleep workshops are currently understudied and the evidence that they are effective to produce long-term positive changes in sleep behaviors and overall sleep in athletes remains unclear (Kaier et al., 2016; O'Donnell and Driller, 2017; Walsh et al., 2021). Sleep workshops could integrate questionnaires such as the ASSQ and ASBQ to assess changes both in sleep quality/quantity but also sleep behaviors. The ASSQ and ASBQ have been in use since 2016 and 2018, respectively; however, few studies have incorporated them to assess sleep difficulty and sleep behaviors in varsity athletes. The ASSQ shows practical utility as a screening mechanism for clinical sleep issues providing a benchmark for follow-up by coaches and athletic training staff (Bender et al., 2018). Further, the ASSQ is reported to have strong test–retest reliability and has been shown to measure change with respect to SDS across pre-competition, competition, and post-competition phases (Bender et al., 2018; Biggins et al., 2021). The ASBQ has recently undergone further psychometric evaluation with recommendations to further assess its factor structure (Miley et al., 2021).

## Conclusion

Validated athlete-specific sleep questionnaires (ASSQ, ASBQ) assessing sleep quantity/quality and sleep behaviors in this sample of varsity athletes representing multiple sports revealed suboptimal sleep hygiene practices pervasive across gender, academic year, and residency contributing to sleep difficulty. Sleep questionnaires like the ASSQ and ABSQ show utility to be integrated into sleep workshops to screen for sleep difficulties and assess sleep hygiene, respectively, and when used in combination may help specifically tailor interventions (e.g., sleep education, screentime, counseling referral, etc.) for individuals and teams, especially if integrated at timely intervals across the academic year. The integration of mindfulness training sessions as part of sleep hygiene workshops could be explored further both in terms of feasibility and effectiveness. Additional studies are warranted to evaluate the ability of the ASSQ and ASBQ to detect meaningful change in sleep quantity/quality and/or sleep behaviors, particularly when assessing the effectiveness of sleep interventions in varsity athletes.

## Data Availability Statement

The raw data supporting the conclusions of this article will be made available by the authors, without undue reservation.

## Ethics Statement

The studies involving human participants were reviewed and approved by Trinity Western University Human Research and Ethics Board. The patients/participants provided their written informed consent to participate in this study.

## Author Contributions

AR, LR, and AC contributed to conception and design of the study. LR organized the database. MB performed the statistical analysis. LR and AR wrote the first draft of the manuscript. MB, AF, and AC wrote sections of the manuscript. All authors contributed to manuscript revision, read, and approved the submitted version.

## Conflict of Interest

The authors declare that the research was conducted in the absence of any commercial or financial relationships that could be construed as a potential conflict of interest.

## Publisher's Note

All claims expressed in this article are solely those of the authors and do not necessarily represent those of their affiliated organizations, or those of the publisher, the editors and the reviewers. Any product that may be evaluated in this article, or claim that may be made by its manufacturer, is not guaranteed or endorsed by the publisher.
